# Pasting alters glycemic index, antioxidant activities, and starch‐hydrolyzing enzyme inhibitory properties of whole wheat flour

**DOI:** 10.1002/fsn3.711

**Published:** 2018-07-16

**Authors:** Stephen A. Adefegha, Tosin A. Olasehinde, Ganiyu Oboh

**Affiliations:** ^1^ Functional Foods and Nutraceuticals Unit Department of Biochemistry Federal University of Technology Akure Ondo State Nigeria; ^2^ Nutrition and Toxicology Division Food Technology Department Federal Institute of Industrial Research Oshodi Lagos Nigeria

**Keywords:** antioxidant, enzymes, glycemic index, Pasting, type 2 diabetes

## Abstract

This study was designed to compare the antioxidant and antidiabetic activities of raw and paste wheat flour. The raw flour was cooked, dried, and milled to obtain the paste flour. The glycemic index, starch, amylose, and amylopectin contents were determined. The inhibitory effects of the raw and paste flour on α‐glucosidase and α‐amylase activities as well as metal‐induced pancreatic damage were also determined. Pasting reduced the glycemic index (63.15%), starch (22.83 g/100 g), amylose (2.88 g/100 g), and amylopectin (17.74 g/100 g) contents. The raw (IC_50_ = 0.50 and 1.20 mg/ml) and paste (IC_50_ = 0.29 and 1.66 mg/ml) flours reduced the activities of α‐amylase and α‐glucosidase, respectively. The paste flour exhibited stronger inhibitory effects against Fe^2+^‐induced pancreatic damage compared to raw flour. The paste flour exhibited better antioxidant and antidiabetic properties and could be a good processing method to improve the medicinal properties of wheat flour.

## 1 INTRODUCTION

Wheat (*Triticum aestivum* L) is a common cereal grain that is widely grown and consumed in different countries (Nair, 2002). Previous reports have shown that wheat has been cultivated in Nigeria for centuries (Oche, 1998). However, local production is very low, and the demands for wheat and wheat‐based products have increased. Wheat is a staple diet which contains carbohydrates, protein, minerals, vitamins, and dietary fiber. The outer part of the grain which is referred to as wheat bran is the in‐digestible portion that aids easy movement of bowels (Nair, 2002). Wheat flour is a coarse‐textured flour ground from the entire wheat kernel and thus contains the bran, germ, and endosperm. It is the main flour source for virtually all confectioneries and bread. Consumption of whole wheat products has been associated with prevention of oxidative stress‐induced diseases such as type 2 diabetes, obesity, hypertension, cardiovascular, and neurological diseases (Kumar et al., 2011). Previous report has shown that biological activities of whole wheat products are linked to the presence of dietary fiber and phytochemicals such as phenolic acids, flavonoids, carotenoids, and tocopherol (Durant, Lenucci, Rescio, Mita, & Caretto, 2012). Furthermore, the evidence from epidemiological study shows an inverse association between whole grain consumption and risk of type 2 diabetes (Jonnalagadda et al., 2011). However, the full understanding of the biochemical rationale has not been established. Furthermore, there are no sufficient data on the effect of processing of the grains on its antidiabetic and antioxidant properties.

Dietary management is an important approach to control postprandial glycemic response, and it could reduce the risk of diabetes and its complications (Adefegha & Oboh, 2012). Some of the factors that affect postprandial glycemic response include glycemic index, food processing, and the presence of some food components. Foods with high GI are known to trigger high glycemic response due to their carbohydrate components. Epidemiological and clinical studies have shown the significance of blood sugar response and the changes in glycemic index (GI) of foods in the management and treatment of diabetes (Eagappan, Philips, & Mohankumar, 2014 **)**. Generally low GI food is considered beneficial due to low postprandial glucose response compared to foods with a high GI. Moreover, there is a correlation between the amylose and amylopectin contents of starchy foods and glycemic index (Behall & Howe, 1995). High sugar, starch, and amylopectin contents of a food can increase its GI (Byrnes, Miller, & Denyer, 1994). Furthermore, previous studies have revealed that some processing techniques can alter the carbohydrate content and medicinal properties of some foods (Jimoh, Adediran, Adebisi, & Biliaminu, 2008). Heat processes can alter the physical form of carbohydrates via gelatinization and retrogradation of starch. Furthermore, heating at high temperature may also increase the viscosity of starch and split starch granules, thereby making it susceptible to enzymatic hydrolysis which can lead to rapid release of monosaccharides such as glucose (Jimoh et al., 2008).

Previous studies have established that oxidative stress contributes to the progression of type 2 diabetes (Oboh, Akinbola, et al., 2015; Valko et al., 2007). Hyperglycemia can induce the production of reactive oxygen species. Furthermore, the pancreas is susceptible to ROS attack, and this may lead to disruption of insulin production and pancreatic islet‐induced oxidative damage (Oboh, Akinbola, et al., 2015). However, antioxidants have been shown to reduce blood glucose level and protect pancreatic cells against oxidative damage. Processing techniques may affect the phenolic content and antioxidant properties of vegetables, fruits, whole grains, and legumes (Adefegha & Oboh, 2011; Oboh, Ademosun, Olasehinde, Oyeleye, & Ehiakhamen, 2015). Although some processing techniques such as cooking, boiling, and roasting have been reported to reduce the phenolic content and antioxidant properties of some foods (Kadiri, 2017; Lemos, Siqueira, Arruda, & Zambiazi, 2012), these same processing methods have been reported to increase the phenolic content, antioxidant, and antidiabetic properties of other foods (Sharma & Gujral, 2011; Oboh, Ademosun, et al., 2015). Pasting is a traditional processing method that is common in West Africa. Flours obtained from cassava, wheat, and yam are usually made into paste and consumed alongside with other kinds of soup. Pasting improves the digestibility of a flour. The pasting process involves reconstituting the wheat flour in a boiling water with continuous stirring. Moreover, as pasting of wheat flour involves the use of heat treatment, this may affect its functional properties and health benefits. To the best of our knowledge, there have been no reports on the effect of pasting on the antioxidant and antidiabetic properties of wheat flour. This study sought to compare the antioxidant activities and inhibitory effects of raw and paste wheat flour on enzymes linked to type 2 diabetes.

## 2 MATERIALS AND METHODS

### 2.1 Materials

α‐glucosidase from rats’ intestine, porcine pancreatic α‐amylase, pepsin, *p*‐nitrophenyl‐α‐D‐glucopyranoside and starch were sourced from (Sigma Chemical Co. St. Louis, MO, USA). 1,10‐phenanthroline, 1,1‐diphenyl–2 picrylhydrazyl (DPPH), Folin–Ciocalteu reagent, deoxyribose, thiobarbituric acid, Tris,3,5‐dinitrosalicylic acid, quercetin, rutin, kaempferol, catechin, epicatechin, gallic, caffeic, and chlorogenic acids were bought from Sigma‐Aldrich, Inc., St Louis, MO. Other chemicals such as aluminum chloride, potassium ferricyanide, sulfuric acid, methanol, hydrogen peroxide, ethanol, Iron (II) sulfate, potassium acetate, sodium dodecyl sulfate (SDS), sodium hydroxide, sodium chloride, potassium chloride, ferric chloride sodium carbonate, sodium acetate, phenol solution, hydrochloric, perchloric, and acetic acids were sourced from (BDH Chemicals Ltd., Poole, UK).

### 2.2 Sample preparation and extraction

Wheat grains were sourced from Akure market in southwest Nigeria and identified at the Department of Biology, Federal University of Technology, Akure. The wheat grains were washed pilled and ground into flour. Raw flour (225 g) was made into paste wheat flour meal which was prepared by continuous stirring of the flour in a pot containing 250 ml of boiling water (100°C) on a hot plate for about 3 min until the paste was formed. After continuous mixing for 3 min, it was cooked to form a smooth thick brown paste. The paste was allowed to dry and ground into flour with a blender. Five (5) grams of the raw and paste flours was weighed separately into a beaker containing 50 ml of distilled water and thoroughly mixed together using a shaker for 2 hr. The mixture was filtered through filter paper (Whatman No 5), and the filtrate was concentrated under pressure using a freeze drier. The extract was reconstituted in distilled water and stored in a refrigerator before it was used for experimental analysis.

### 2.3 Determination of total phenol content

The method of Singleton, Orthofer, and Lamuela‐Raventos (1999) was used to determine the total phenol content of the raw and paste flours, respectively. The extract (5 mg/ml) was oxidized with 2.5 ml 10% Folin–Ciocalteau reagent (v/v) which was neutralized by 2.0 ml of 7.5% Na_2_CO_3_. The mixture was incubated for 40 min at 45 °C, and the absorbance was measured at 765 nm using a spectrophotometer. Gallic acid was used as a standard, and the total phenol content was subsequently calculated as gallic acid equivalent.

### 2.4 Determination of total flavonoid content

The reaction mixture consists of 0.5 ml of the aqueous extract (5 mg/ml) derived from the flour samples, absolute methanol (0.5 ml), 10% AlCl_3_ (50 μl), 1 M potassium acetate (50 μl), and H_2_O (1.4 ml). The mixture was incubated at room temperature for 30 min. Quercetin was used as standard. The absorbance of the mixture was measured at 415 nm. The total flavonoid content was subsequently calculated as quercetin equivalent (Meda, Lamie, Romito, Millogo, & Nacoulma, 2005).

### 2.5 High‐performance liquid chromatography–Diode array detector (HPLC‐DAD) analysis

HPLC‐DAD was used to determine and quantify the phenolic compounds present in the raw and wheat paste flour according to the method of Oboh, Ademiluyi, et al. (2015). Extracts (40 μl) obtained from the raw and paste wheat samples (10 mg/ml) were injected via the autosampler (model SIL‐20A Shimadzu) of the HPLC system. Phenomenex C_18_ column (4.6 mm × 250 mm × 5 μm particle and mobile phase [water with 1% formic acid (v/v) (solvent A) and HPLC grade methanol (solvent B)] were used in the analysis. The flow rate and injection volume were set at 0.6 ml/min 40 μl, respectively. The composition gradient was 5% solvent B reaching 15% at 10 min; 30% solvent B at 25 min, 65% solvent B at 40 min, and 98% solvent B at 45 min, followed by 50 min at isocratic elution until 55 min. The sample and mobile phase were filtered in a membrane filter (0.45 μm). Reference standards such as quercetin, rutin, catechin, epicatechin, kaempferol, gallic acid, caffeic acid, chlorogenic acid, and quercitrin (0.025–0.300 mg/ml) were prepared by dissolving the compounds in a mixture of methanol: water (1:1, v/v). Quantifications were carried out by integration of the peaks using the external standard method, at 280 nm for catechin and epicatechin, 325 nm for chlorogenic acid, and 366 for rutin, quercetin, and kaempferol. Confirmation of the peaks was made by comparing the retention time of individual peaks with the standards and DAD spectra with wavelengths ranging from 200 to 600 nm. The analysis was carried out at ambient temperature and in triplicates.

### 2.6 Determination of starch and sugar contents

The samples (0.02 g) were dissolved in 80% hot ethanol to extract sugar and starch. The solution was centrifuged for 10 min at 200 rpm, and the supernatant (S1) was obtained and used for free sugar analysis (Onitilo, Sanni, & Daniel, 2007). To determine free sugar content, an aliquot of the supernatant (0.2 ml) was added to a mixture of 5% phenol solution (0.5 ml) and conc. H_2_SO_4_ (2.5 ml). After the mixture was cooled, the absorbance was measured at 490 nm. The residue was hydrolyzed with perchloric acid (7.5 ml) for 1 h, and the solution was diluted with distilled water in a 25‐mL flask. The mixture was then filtered through filter paper (Whatman No 2). The filtrate (0.05 ml) obtained was mixed with 0.5 ml of phenol solution (5%) and 2.5 ml conc. H_2_SO_4_. The absorbance of the solution was measured after it was cooled. The total free sugar and starch contents were obtained by calculation from a glucose standard curve.

### 2.7 Determination of amylose and amylopectin contents

Briefly, ethanol (95%, 1 ml) and 9.2 mL NaOH (1 N) were added to the flour samples (0.1 g) in different tubes and were heated in a water bath for 10 min. The solution was allowed to cool after which an aliquot was added to 1 N acetic acid (0.1 ml) and iodine solution (0.2 ml). Water was added to the mixture to make the solution up to 10 ml. The color of the solution was developed after it was mixed and left for 20 min. The absorbance of the mixture was measured at 620 nm (Adedayo, Oboh, Oyeleye, & Olasehinde, 2016). Amylose content of the flour samples was determined from the calibration curve using amylose standard. The amylopectin contents of the flour samples were determined using the formula below:

Amylopectin=starch value−amylose value

### 2.8 Determination of glycemic index

The sample (25 mg) was dissolved in 10 ml buffer solution (0.1 M HCl‐KCl buffer) containing 1 mg pepsin. The solution was incubated in a shaker bath at 40°C for 30 min. Phosphate buffer pH 6.9 and 2.5 ml of α‐amylase solution (0.005 g in 10 ml) were added to the solution after which was incubated at 37°C. The digest (200 μl) was placed into a test tube at 30 min interval. (0, 30, 60, 90, 120, 150, and 180 min). Different portion of the digest was boiled for 15 min, and 500 μl of 0.4 M of sodium acetate (pH 4.75) and 5 μl of α‐glucosidase solution (0.005 g in 10 ml) were added to each solution. The mixtures were incubated for 45 min at 60°C. DNSA solution (200 μl) was added to the tube, and the mixture was heated for 5 min at 100°C. Distilled water (2 ml) was added to the mixture and was centrifuged at 3000 rpm for 5 min. The supernatants were decanted, and the absorbance was measured at 540 nm. The sum of area under the curve for each sample was divided by the sum of area under the curve for standard glucose and multiplied by 100. The value obtained is the glycemic index (Brouns et al., 2005).

### 2.9 α‐amylase activity assay

The effect of the flour samples on α‐amylase activity was assessed using the method of Bernfield (1951). The reaction mixture which contains different concentration of each flour samples (0.4–1.6 mg/ml) and 500 μl of Hog pancreatic α‐amylase (EC 3.2.1.1) (0.5 mg/ml) was incubated at 25°C for 10 min. Five hundred microliter of starch (1%) solution prepared with 0.02 M sodium phosphate buffer (pH 6.9) was added to the previous mixture and was incubated for 10 min at 25°C. Dinitrosalicylic acid (1.0 ml) was added to the mixture to stop the reaction. The resultant solution was incubated for 5 min at 100 °C. The solution was allowed to cool, and distilled water was added to the mixture before the absorbance was measured at 540 nm. α‐amylase activity was calculated and expressed as percentage inhibition using the following formula:

(1) $$\begin{array}{cc} \%\text{Inhibition}= & \left[\left({\text{Abs}}_{\mathrm{Control}}-{\text{Abs}}_{\mathrm{Samples}}\right)/{\text{Abs}}_{\mathrm{Control}}\right]\\ & \times 100\;\text{where Abs is absorbance} \end{array}$$

### 2.10 α‐glucosidase activity assay

α‐glucosidase inhibitory activity of the samples was determined according to the method of Adefegha and Oboh (2012). The enzyme (1.0 U/ml) was prepared in sodium phosphate buffer (0.1 M, pH 6.9). The extracts (0.4–16 mg/ml) were added to the enzyme (100 μl) which was incubated for 10 min at room temperature. Fifty microliter of *p*‐nitrophenyl‐α‐D‐glucopyranoside [5 mM of the substrate was dissolved in phosphate buffer (0.1 M, pH 6.9)] was added to the mixture. The solution was incubated at room temperature for 5 min. After the incubation, the absorbance was measured at 405 nm. The α‐glucosidase inhibitory activity was expressed as percentage inhibition using equation (1).

### 2.11 Experimental animals

Male Wistar albino rats (180 g) were used in this study specifically for the lipid peroxidation assay. The rats were fed with standard feed and water prior to the experiment. The Guide for Care and Use of Laboratory Animals published by the National Institute of Health (USA) (Public Health Service, 1996) was strictly followed.

#### 2.11.1 Lipid peroxidation assay

The rat was sacrificed, and the pancreas was rapidly dissected and placed on ice. The tissue was weighed and homogenized in cold saline and centrifuged at 3000 g for 10 min. The supernatant (S1) was decanted and used for the assay. The reaction mixture contained S1 fraction (100 μl), 30 μl of 0.1 M Tris–HCl buffer (pH 7.4), sample (0–100 μl), and 30 μl of FeSO_4_ (250 μM). Three hundred microliter of water was added to the mixture before it was incubated at 37°C for 1 hr. Three hundred microliter of SDS (8.1%) was added to the mixture to develop a color. Furthermore, 500 μl of acetic acid/HCl (pH 3.4) and 500 μl of 0.8% thiobarbituric acid (TBA) were also added to the mixture. The mixture was incubated at 100°C for 1 hr. The absorbance was measured at 532 nm, and malondialdehyde levels were calculated as percentage control (Ohkawa, Ohishi, & Yagi, 1979).

### 2.12 Fe^2+^ chelation assay

The ability of the extracts to chelate Fe^2+^ was determined using the method of Puntel, Nogueira, and Rocha (2005). Freshly prepared 500 μM FeSO_4_ (150 μl) was added to a reaction mixture containing 168 μl 0.1 M Tris–HCl (pH 7.4), 218 μl saline (0.8%), and the extracts (0.25–1.0 mg/ml). The reaction mixture was incubated for 5 min before the addition of 13 μl of 0.25% 1, 10‐phenanthroline (w/v). The absorbance was subsequently measured at 510 nm in a spectrophotometer. Fe^2+^ chelating ability was calculated and expressed as percentage.

### 2.13 DPPH radical scavenging assay

DPPH (1,1‐diphenyl–2 picrylhydrazyl) radical scavenging ability of the flours was determined according to Gyamfi, Yonamine, and Aniya (1999). The extract (1 ml) was added to 1 ml 0.4 mM DPPH radicals (prepared with methanol). The resultant solution was placed in the dark, and the absorbance was measured after 30 min at 516 nm. The control experiment was carried out using 2 ml DPPH solution without the test samples. The DPPH radical scavenging ability was subsequently calculated as percentage control.

### 2.14 Hydroxyl radical scavenging assay

The method of Halliwell and Gutteridge (1981) was used to evaluate the capacity of the extracts to scavenge OH radicals. The reaction mixture consists of the extract (0–100 μl), 20 mM deoxyribose (120 μl), 0.1 M phosphate buffer (400 μl), 500 μM of FeSO_4_ (40 μl), and distilled water (800 μl). The mixture was incubated in a water bath for 30 min at 37°C. After the incubation, 2.8% trichloroacetic acid (0.5 ml) was added to the mixture to stop the reaction. Finally, 0.4 ml of thiobarbituric acid (0.6%) was added to the mixture. The mixture was heated at 100°C for 20 min. The absorbance of the resultant solution was measured at 532 nm.

### 2.15 Statistical analysis

Statistical significance was established using one‐way analysis of variance, and data were reported as mean ± standard error using Graph pad prism 6.0. Significant difference was accepted at *p *≤ 0.05, and IC_50_ values were calculated using nonlinear regression analysis.

## 3 RESULT AND DISCUSSION

### 3.1 Phenolic composition of raw and paste flour

**Figure 1:**
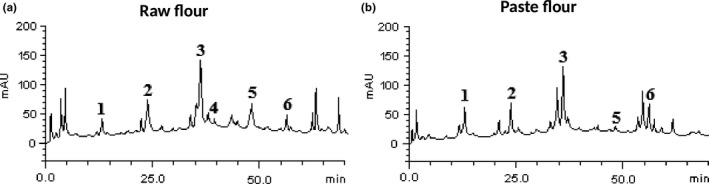
Representative high performance liquid chromatography profile of wheat raw and paste flour. Catechin (peak 1), chlorogenic acid (peak 2), rutin (peak 3), epicatechin (peak 4), quercetin (peak 5) and kaempferol (peak 6)

Figure 1 revealed the phenolic constituents of the raw and paste wheat flour. The major compounds detected in the flours consist of one phenolic acid and five flavonoids. Chlorogenic acid, catechin, rutin, quercetin, and kaempferol were present in the raw and paste flours. This result correlates with the report of Sanak, Allam, and El‐Shazely (2016), although the authors reported the presence of more phenolic acids and flavonoids in *T. aestivum* and *T. durum*. Furthermore, Laddomada et al. (2015b); Laddomada et al. (2015b) reported the presence of ferulic, 4‐dihydroxybenzoic, syringic, p‐coumaric, vanillic, and sinapic acids but did not report the presence of flavonoids in different cultivars of Italian wheat flour meal. In this present study, there was no significant difference in chlorogenic acid levels of the raw (1.95 mg/g) and paste (1.94 mg/g) flours as shown in Table 1. The level of epicatechin (0.08 mg/g) in the raw flour was low; however, there was no trace of this compound in the paste flour. Our findings suggest that the heat treatment involved in the pasting process may contribute to the disappearance of epicatechin in the paste flour as shown in Table 1. This result agrees with the findings of Payne, Kenneth, Craigrank, and Stuart (2010) which revealed a complete loss of epicatechin due to roasting. Similarly, Table 1 revealed that pasting caused a significant decrease in the levels of rutin (6.05 mg/g) and quercetin (0.11 mg/g). The decrease in rutin and quercetin levels could be attributed to the cooking temperature during pasting. Sharma and Lee (2016) reported a decrease in quercetin in onions due to prolong heating. On the contrary, pasting caused a significant increase in the levels of catechin (1.82 mg/g) and kaempferol (1.79 mg/g) as presented in Table 1. The increase in catechin levels could be attributed to the release of bound catechins within the food matrix during heat treatment. The phenolic and flavonoid contents of the raw and paste flours are shown in Table 2. Pasting significantly increased the phenolic content of the flour (paste flour = 49.88 mg GAE/g; raw flour = 42.37 mg GAE/g). The increase in phenolic content of the paste flour agrees with previous findings on changes in the chemical constituents of some fruits and vegetables due to different processing techniques (Adefegha & Oboh, 2011). However, the flavonoid content of the raw (35.71 mg QE/g) and paste flour (36.42 mg QE/g) was not significantly different as shown in Table 2. This shows that some of the flavonoids present in the paste flours were not affected by the treatment.

**Table 1:** Phenolic constituents of wheat raw and wheat paste

Parameter	Raw flour mg/g	Paste flour mg/g
Catechin	0.63 ± 0.02^a^	1.82 ± 0.01^b^
Chlorogenic acid	1.95 ± 0.01^b^	1.94 ± 0.04^b^
Rutin	6.34 ± 0.05^c^	6.05 ± 0.02^b^
Epicatechin	0.08 ± 0.04^d^	ND
Quercetin	1.73 ± 0.01^e^	0.11 ± 0.01^c^
Kaempferol	0.59 ± 0.01^a^	1.79 ± 0.03^b^

Notes

Value represents mean ± standard deviation of replicates (*n *= 3). Values with different superscript letter on the same row are significantly different (*p* < 0.05).

ND: Not detected.

**Table 2:** Total phenol (mg/g), flavonoid content (mg/g), starch content (g/100 g), sugar content (g/100 g), starch/sugar ratio, glycemic index (%), amylose content (g/100 g), amylopectin content (g/100 g), and the amylose/amylopectin ratio of wheat flour (*Triticum* spp.)

Parameter	Raw flour	Paste flour
Total phenol (GAE mg/g)	42.37 ± 1.75^a^	49.88 ± 1.46^b^
Total flavonoid (QE mg/g)	35.71 ± 0.87^b^	36.42 ± 1.33^b^
Starch (g/100 g)	24.99 ± 0.67^a^	22.83 ± 0.80^b^
Sugar (g/100 g)	3.68 ± 0.05^a^	3.29 ± 0.03^b^
Starch/sugar ratio	0.15	0.14
Amylose content (A) (g/100 g)	3.63 ± 0.60^a^	2.88 ± 0.83^b^
Amylopectin content (Am) (g/100 g)	21.36 ± 1.25^a^	19.95 ± 1.67^a^
A/Am	0.19	0.16
GI (%)	71.92 ± 1.25^a^	63.15 ± 1.33^b^

Note

Value represents mean ± standard deviation of replicates (*n *= 3). Values with different superscript letter along the same row are significantly different (*p* < 0.05).

### 3.2 Starch and sugar contents

The starch and sugar contents of raw flour were 24.99 g/100 g and 3.68 g/100 g, while that of paste flour was 22.83 g/100 g and 3.29 g/100 g, respectively, as presented in Table 2. The observed reduction in the sugar and starch contents of the paste flour could be due to the fact that carbohydrates are not stable during exposure to thermal processes. Several reports have established that sugar content of starchy foods may reduce during processing (Camire, Camire, & Krumhar, 1990; Singh, Gamlath, & Wakeling, 2007). This could be due to the conversion of sucrose to reducing sugars such as glucose and fructose which may be lost during Maillard reactions with amino acids leading to the formation of melanoidins. Heat treatment has been reported to reduce sugar levels in some foods due to the formation of Maillard reaction products which are formed by the reaction of sugars and amino acids (Sharma et al., 2015). The result revealed that paste flour had lower starch and sugar contents than the raw flour. Previous reports have shown that processing of foods may contribute to the development of some diet‐related diseases such as diabetes and atherosclerosis (Birlouez‐Aragon et al., 2010). However, some scientific investigations have revealed that processing techniques such as roasting, blanching, boiling, cooking, and pasting can influence the nutritional and antioxidant properties of some foods (Oboh, Akinbola, et al., 2015; Valko et al., 2007). In this study, we observed that pasting reduced the sugar and starch contents of wheat flour. This result correlates with the report of Apata (2008) which revealed a decrease in starch and sugar contents of some legumes. High levels of starch and sugar consumption may increase blood glucose response in diabetic individuals. Hence, the observed decrease in starch and sugar levels of the paste flour could be beneficial as it would reduce postprandial blood glucose compared to the raw wheat flour.

### 3.3 Amylose and amylopectin contents

Dietary starch contains mixtures of amylose and amylopectin in varying proportions. Moreover, amylose and amylopectin ratio in food has significant effect on postprandial responses (Lafiandra, Riccardi, & Shewry, 2014). Starchy foods with high amylose content have been reported to trigger low blood glucose and insulin response compared to same foods with high amylopectin content. Table 2 reveals the amylose and amylopectin ratio raw and wheat paste flour. Pasting reduced the amylose and amylopectin ratio of the flour. Moreover, this result correlates with the report of Noranizan, Dzulkifly, and Russly (2010) which revealed that thermal treatment reduced the amylose and amylopectin ratio of wheat starch at 110°C. As shown in Table 2, amylose content of the raw flour (3.63 g/100 g) was significantly higher than the paste flour (2.88 g/100 g). The amylopectin content of the paste flour (19.95 g/100 g) varies significantly than the raw flour (21.36 g/100 g). Our findings show that pasting significantly reduced the amylose and amylopectin contents of wheat flour. Nevertheless, the amylopectin content of the flour was significantly higher than the amylose content. The open branched structure of starches with large proportions of amylopectin makes it susceptible to carbohydrate‐hydrolyzing enzymes and can be digested and absorbed rapidly into the blood (Wiseman, Higgins, & Denyer, 1996). However, the straight chain of glucose unit makes amylose more compatible and difficult to digest rapidly, thereby slowing down the absorption of glucose. Previous investigation has revealed that rapid breakdown of starches with large proportions of amylopectin contributes to the development of insulin resistance and diabetes (Byrnes et al., 1994). Wiseman et al. (1996) reported that rats fed with high amylose diet had low glycemic response and no significant change in insulin resistance. The decrease in the amylopectin and amylose contents caused by pasting could be attributed to the fact that the paste meal was dried after cooking and ground into flour. Furthermore, the exposure of the paste flour to the heat treatment during pasting may trigger starch hydrolysis which could lead to the release of reducing sugars that can react with amino acids in Maillard reaction, hence reducing the amylose and amylopectin contents of the flour.

### 3.4 Glycemic index

Previous reports have revealed the relationship between amylose/amylopectin ratio and glycemic index of foods rich in carbohydrate (Behall & Howe, 1995). The consumption of high GI foods can increase the risk of diabetes. However, low GI foods can reduce glycemic response. The glycemic index of the paste flour as shown in Table 2 was 63.15% while that of the raw flour was 71.92%. Moreover, the GI of paste wheat flour (63.15%) was lower than what was reported by Reyes‐Pérez, Salazar‐García, Romero‐Baranzini, Islas‐Rubio, and Ramírez‐Wong (2013). Furthermore, Foster‐Powell, Holt, and Brand‐Miller (2002) reported the GI of cooked Durum wheat that was cooked for 10 and 20 min to be 50 and 52%, respectively.

This result shows that pasting significantly reduced the glycemic index of the flour. Using glucose as reference, foods have been classified as low GI (0–55) medium GI (56–69) and high GI (≥70). Regarding this classification, the paste flour has a medium GI while the raw flour had a high GI. The high GI obtained from the raw wheat flour can be attributed to its high amylopectin content. This is due to the fact the amylopectin chain is susceptible to enzymatic attack which could lead to rapid release of glucose into the blood. However, pasting reduced the GI significantly in the paste flour. Similar result was reported by Jimoh et al. (2008) on the decrease in GI of yam paste meal. There are indications that cooking of the flour may increase the GI because starch become swollen, gelatinized, and are rapidly digested during the heat process. However, cooling of the paste, drying, and milling into flour may reverse its gelatinization, thereby making it more resistant to starch hydrolysis. In addition, the formation of melanoidins in Maillard reaction during cooking could also reduce the GI of the paste flour. The browning products of the Maillard reaction could inhibit the enzymes involve in starch digestion, thereby disrupting the release of glucose (Adedayo, Ademiluyi, Oboh, & Akindahunsi, 2012). Therefore, the low GI observed in the paste flour could be ascribed to the nonenzymatic browning products which were formed during pasting. High GI foods can induce hyperglycemia and could contribute to the development of diabetes. However, control of high blood glucose involves inhibition of carbohydrate‐hydrolyzing enzymes. Inhibition of these enzymes is a good strategy to influence the GI of foods and could be useful for the management and/or treatment of diabetes.

### 3.5 Antidiabetic activity

**Figure 2:**
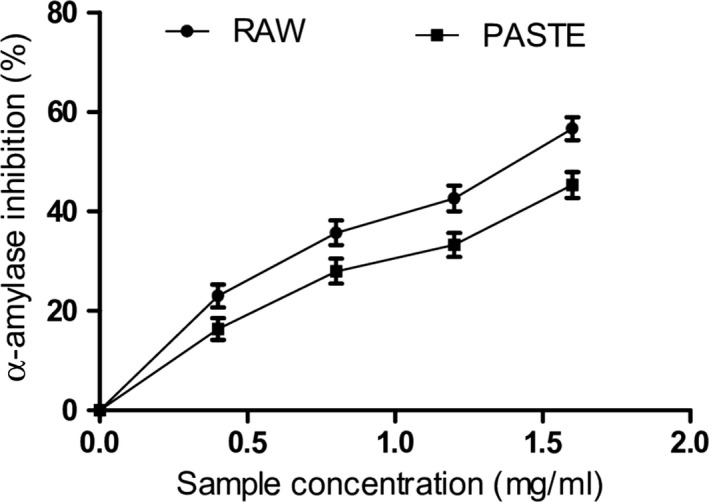
α‐amylase inhibitory activity of raw and paste wheat flour

**Figure 3:**
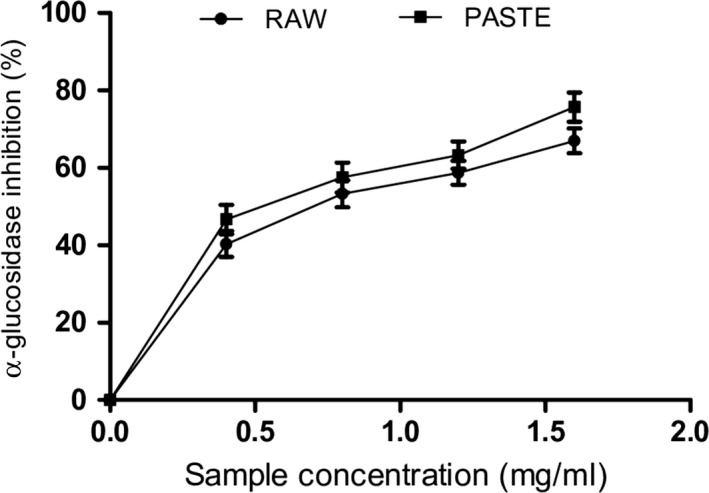
α‐glucosidase inhibitory activity of raw and paste wheat flour

The control of postprandial blood glucose in diabetic conditions may prevent hyperglycemia and progression of diabetes. Inhibition of carbohydrate‐hydrolyzing enzymes such as α‐amylase and α‐glucosidase has been established as a therapeutic strategy for the reduction in postprandial hyperglycemia. α‐amylase is involved in the breakdown of starch into disaccharides and oligosaccharides while intestinal α‐glucosidase catalyzes the hydrolysis of glucose which is absorbed into the bloodstream. However, the inhibition of these enzymes will reduce the absorption of glucose into the bloodstream, thereby preventing hyperglycemic events in diabetic conditions. Our findings revealed that extracts from raw and paste wheat flours reduced α‐amylase and α‐glucosidase activities as shown in Figures 2 and 3, respectively. The raw flour (IC_50_ = 1.34 mg/ml) had significantly higher α‐amylase inhibitory activity than paste flour (IC_50_ = 1.69 mg/ml). Moreover, extract from the paste flour (IC_50_ = 0.92 mg/ml) exhibited higher α‐glucosidase inhibitory activity compared to the raw flour (IC_50_ = 1.01 mg/ml) (Table 3). The observed decrease in α‐amylase activity could be due to the retardation of the breakdown of complex carbohydrates by α‐amylase to form oligosaccharides which are further hydrolyzed into glucose by intestinal α‐glucosidase (Adefegha & Oboh, 2012). However, paste wheat flour had significantly higher inhibitory effect on α‐glucosidase activity compared to the raw flour. High α‐glucosidase inhibition is important and effective for the management of type 2 diabetes compared to α‐amylase inhibition (Ademiluyi & Oboh, 2013). Moreover, excessive inhibition of α‐amylase is associated with several side effects. Although both flours reduced the activities of α‐amylase and α‐glucosidase, our findings suggest that paste flour exhibited stronger antidiabetic potentials compared to raw flour. The α‐amylase and α‐glucosidase inhibitory activities of the wheat flour could be attributed to their phenolic and flavonoid contents. Phenolic compounds such as rutin, quercetin, catechins, and chlorogenic acid have been shown to be potent inhibitors of α‐amylase and α‐glucosidase activities, thus reducing postprandial hyperglycemia (Adefegha, Oboh, Oyeleye, & Osunmo, 2016). Structure–activity relationship studies have shown that the catechol group in the B‐ring of quercetin and kaempferol contributes immensely to their α‐glucosidase inhibitory activity (Proenca et al., 2017). Moreover, quercetin has been reported to bind extensively with its hydrogen atoms to the reactive pocket of the enzyme, thereby increasing its inhibitory activity (Proenca et al., 2017). Phenolic compounds are safer than synthetic inhibitors with a greater advantage due to their ability to inhibit hyperglycemia and oxidative stress (Adefegha & Oboh, 2012). These results revealed that pasting did not hinder the inhibitory effect of the extract on the enzymes although a slight reduction was observed in the inhibition of α‐amylase activity compared to the effect of the raw wheat flour. Furthermore, the formation of melanoidins (browning products) in Maillard reaction during cooking could be responsible for the high α‐glucosidase inhibition observed in the paste flour (Adedayo et al., 2012).

**Table 3:** IC_50_ values of inhibition α‐amylase α‐glucosidase activities, DPPH, OH radical scavenging, and Fe^2+^‐chelating abilities of raw and paste wheat flour

Samples	Raw	Paste
α‐amylase (mg/ml)	1.34 ± 0.03^b^	1.69 ± 0.03^a^
α‐glucosidase (mg/ml)	1.01 ± 0.03^a^	0.92 ± 0.02^b^
MDA	2.20 ± 0.02^a^	1.80 ± 0.04^a^
DPPH (mg/ml)	2.46 ± 0.03^b^	1.48 ± 0.02^a^
OH (mg/ml)	0.77 ± 0.05^a^	0.56 ± 0.08^b^
Fe^2+^ chelation (mg/ml)	0.81 ± 0.04^a^	0.60 ± 0.03^b^

Note

Values represent mean ± standard deviation, number of sample replicate *n *= 3. Values with different superscript letter along the same row are significantly different (*p* < 0.05).

### 3.6 Antioxidant activity

**Figure 4:**
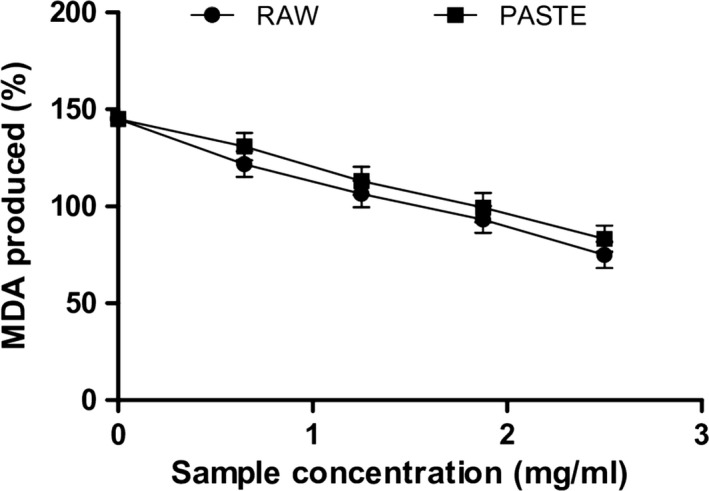
Inhibition of Fe^2+^‐induced lipid peroxidation in rat's pancreas by raw and paste wheat flour

Hyperglycemia can trigger the formation of free radicals in patients with diabetes and this can contribute to the progression of type 2 diabetes and diabetic complications such as cardiovascular diseases, diabetic neuropathy, and nephropathy (Valko et al., 2007). In this study, Fe^2+^significantly induced malondialdehyde (MDA) production in pancreas homogenates as shown in Figure 4. However, raw and paste flours (0.65–2.5 mg/ml) significantly (*p* < 0.05) reduced MDA levels [raw flour (121.77%–74.98%) and paste flour (130.84%–83.33%)] in pancreas homogenates. Paste flour (1.80 mg/ml) exhibited stronger inhibitory effect on MDA production compared to raw flour (2.20 mg/ml) as shown by their IC_50_ values in Table 3. This result agrees with the report of Oboh and Adefegha (2010) on the inhibition of Fe^2+^‐induced lipid peroxidation by wheat biscuit. Iron overload in pancreatic cells can lead to pancreatic β‐cell damage, deficiency of insulin, and decreased glucose uptake. This is because free Fe can react with hydrogen peroxide to form hydroxyl radicals which can induce lipid peroxidation, protein oxidation, and DNA damage in pancreatic cells (Oboh & Adefegha, 2010). Therefore, the inhibition of Fe^2+^‐induced MDA production in rats’ pancreas by the raw and paste flour may prevent oxidative damage to the pancreas.

**Figure 5:**
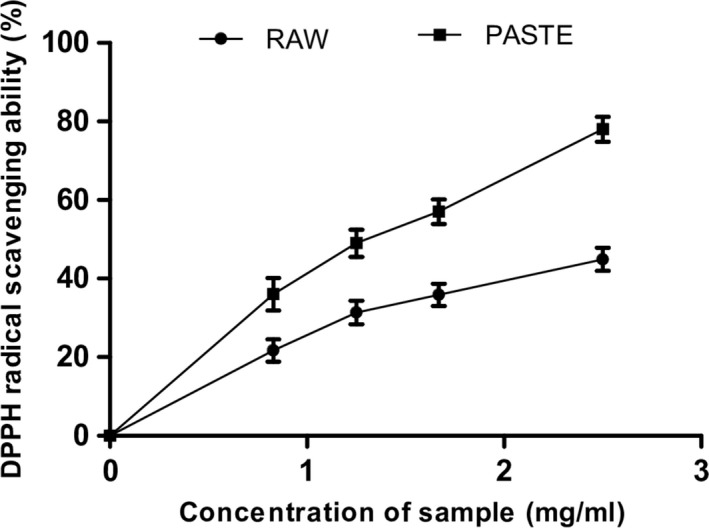
DPHH radical scavenging activity of raw and paste wheat flour

**Figure 6:**
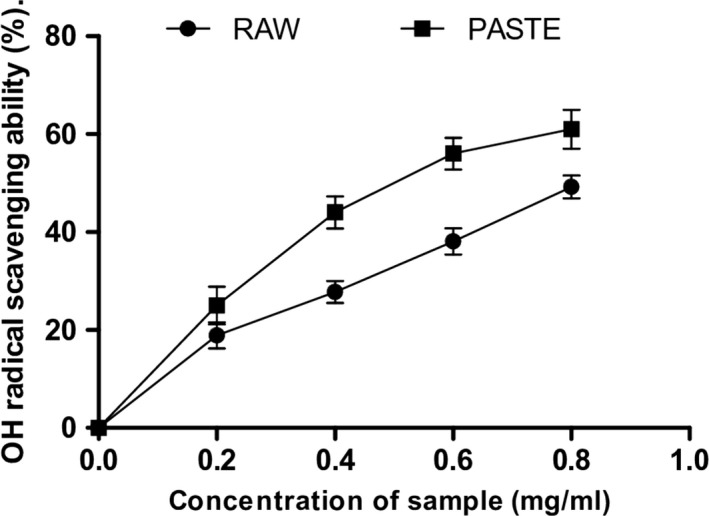
OH radical scavenging activity of raw and paste wheat flour

**Figure 7:**
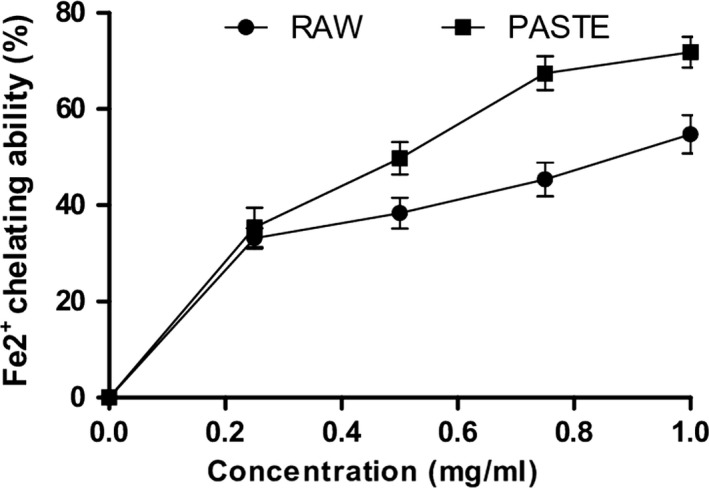
Fe^2+^ chelating ability of raw and paste wheat flour

Furthermore, both flours scavenged DPPH, OH radicals and chelated Fe^2+^ as shown in Figures 5, 6, 75‐7. The IC_50_ values of the radical scavenging and Fe^2+^‐chelating abilities of the flours are presented in Table 3. The DPPH radical scavenging ability of the flours revealed that raw flour (IC_50_ = 0.48 mg/ml) had significantly (*p* < 0.05) higher scavenging ability than the paste flour (IC_50_ = 1.02 mg/ml). Similarly, the raw flour (IC_50_ = 410 μg/ml) also had significantly (*p* < 0.05) higher OH radical scavenging ability than the paste flour (IC_50_ = 520 μg/ml). Furthermore, paste flour (IC_50_ = 0.20 mg/ml) had strong chelating ability compared with raw flour (IC_50_ = 0.39 mg/ml). Pasting significantly increased the chelating ability of the flour. The DPPH, OH radical scavenging activities, and Fe^2+^‐chelating ability of the flours revealed that they contain antioxidants and can prevent radical‐induced oxidative stress in rats’ pancreas. Our findings also revealed that pasting increased the antioxidant activities of the wheat flour. Thermal treatment increased the levels of catechin and kaempferol in the paste flour and this may contribute to its higher antioxidant activity compared to the raw flour. The high radical and metal chelating activities of the paste flour could be associated with high levels of catechin and kaempferol present in the extract compared to the raw wheat flour. Furthermore, the formation of melanoidins in the paste flour may also contribute to the high radical and metal chelating activities of the extracts. Previous studies have shown that melanoidins exhibit strong antioxidant activities due to their capacity to donate hydrogen ions and the presence of catechol moieties in their structure (Adedayo et al., 2012; Martín et al., 2009). Further investigation is required to identify and isolate the melanoidins formed in the paste flour which may be responsible for its antioxidant and enzyme inhibitory activities.

## 4 CONCLUSION

This study revealed that pasting reduced amylose/amylopectin content, glycemic index, and increased the total phenolic content, antioxidant, and antidiabetic properties of wheat flour. The antidiabetic properties of the flours were attributed to their ability to inhibit α‐amylase and α‐glucosidase activities and prevent MDA‐induced pancreatic damage. Our findings show that paste flour could be more beneficial than raw flour in terms of glycemic response and management of diabetes.

## ETHICAL STATEMENT

The ethics committee of the School of Science, Federal University of Technology, Akure, approved the use of animals in this study. The National Institute of Health's Guide for Care and Use of Laboratory Animals was also followed in this study.

## CONFLICT OF INTEREST

All the authors declare no conflict of interest.
